# Nucleolar protein 6 as a potential oncogenic factor in colorectal cancer

**DOI:** 10.1371/journal.pone.0340047

**Published:** 2026-02-02

**Authors:** Shunxin Song, Yixin Zhang, Lingxi Li, Kaikai Zhou, Yuguo Zhao, Zhao Yang, Shuohao Guo, Rongzhang He, Jianwen Zhang

**Affiliations:** 1 Department of General Surgery, The First People’s Hospital of Chenzhou, University of South China, Chenzhou, People’s Republic of China; 2 Translational Medicine Institute, The First People’s Hospital of Chenzhou, University of South China, Chenzhou, People’s Republic of China; 3 Department of Colorectal Surgery, The First People’s Hospital of Chenzhou, University of South China, Chenzhou, People’s Republic of China; Chung Shan Medical University, TAIWAN

## Abstract

**Background:**

Colorectal cancer (CRC) is a common malignancy of the digestive tract associated with high mortality rates and significant invasive properties. Despite advancements in research, a comprehensive understanding of the regulatory mechanisms underlying CRC remains elusive.

**Objective:**

This study aimed to investigate the potential role of nucleolar protein 6 (NOL6) and its related genes as novel biomarkers for cell proliferation in CRC. The findings of this study could significantly contribute to early diagnosis and more effective therapeutic strategies for CRC.

**Methods:**

Human CRC cell line HCT116 was cultured under standard conditions. Quantitative real-time polymerase chain reaction and immunohistochemistry analysis were used to measure NOL6 expression levels in CRC tissues. Cell proliferation was assessed using the MTT assay, Celigo cell count assay, and colony formation assays, while flow cytometry was employed to evaluate cell apoptosis. Additionally, a transwell migration assay was performed to evaluate CRC cell migration and invasion. Comprehensive proteomic and transcriptomic analyses were performed to identify the downstream genes and pathways affected by NOL6 knockdown. The expression of these genes was further validated by Western blotting. Xenograft mouse models were used to determine the effects of NOL6 on CRC *in vivo*. Tandem mass tags (TMT)-labeled quantitative proteomic technology and bioinformatic analysis were employed to identify the functional pathway and proteins regulated by NOL6.

**Results:**

The Cancer Genome Atlas data analysis revealed a significant upregulation of NOL6 in CRC cells compared with adjacent normal cells. In HCT116 cells, downregulation of NOL6 was associated with decreased proliferation and colony formation, as well as increased apoptosis. Additionally, NOL6 knockdown resulted in a decrease in the weight and volume of tumors in nude mice, suggesting its role in tumorigenesis. TMT and Western blot analyses revealed that NOL6 knockdown suppressed MCM3 and MCM7 expression.

**Conclusion:**

This study demonstrated that NOL6 functions as an oncogene that facilitates CRC progression, suggesting its potential role as a therapeutic target for CRC management.

## Background

Colorectal cancer (CRC) is a common malignancy of the digestive tract and the third leading cause of cancer-related mortality globally. Annually, over 1.9 million new cases are diagnosed, resulting in approximately 900,000 deaths [1,2]. In China, rectal cancer accounts for approximately 50% of all CRC cases, which is significantly higher than the approximately 30% reported in Western countries [3]. Despite significant advancements in cancer prevention and treatment that have considerably improved the 5-year survival rate of CRC patients in China [4,5], the prognosis for these patients remains poor due to late diagnosis and treatment. Early diagnosis and treatment of CRC at a curable stage are crucial for reducing mortality rates [4,6,7]. Recent advancements in elucidating the pathogenesis and molecular mechanisms underlying CRC provide insights for early detection and diagnosis [8]. Although several nucleolar genes, including SNHG8, SNHG1, and NIFK, have been implicated in CRC development [9–11], the specific gene network associated with its development remains largely undefined, with many unidentified genes.

The nucleolus is the site of rRNA gene storage, rRNA synthesis and processing, and ribosomal subunit assembly. An adequate supply of ribosomes is essential for efficient protein biosynthesis and rapid cell proliferation in cancer cells [12–14]. Consequently, nucleolar proteins have emerged as potential targets for anticancer therapies.

Nucleolar protein 6 (NOL6), located on chromosome 9p13 and spanning 11,434 base pairs, consists of 26 relatively short exons and 25 introns [15]. It is also known as nucleolar RNA-associated protein (NRAP) and is highly conserved across species [13,15]. In preliminary studies, we observed that NOL6 is overexpressed in human CRC, suggesting its potential as a nucleolar protein target for human CRC.

In this study, we investigated the role of NOL6 in CRC both *in vitro* and *in vivo* and hypothesized its potential role as an oncogene in CRC. We also determined the effects of NOL6 knockdown on proliferation and apoptosis in CRC cells, proposing that NOL6 knockdown inhibits proliferation and arrests cell-cycle. The specific mechanisms underlying these effects were elucidated using tandem mass tags (TMT) labeling and analysis. Our findings demonstrate that NOL6 functions as an oncogene that facilitates CRC progression, highlighting its potential as a therapeutic target.

## Materials and methods

### CRC patient samples

CRC and adjacent para-carcinoma tissues were obtained from 97 CRC patients who underwent surgery at the Department of General Surgery, the First People’s Hospital of Chenzhou from 2024.12.1–2025.5.30. This study was approved by the Ethics Committee of the First People’s Hospital of Chenzhou (2024109), and written informed consent was obtained from all participants to ensure they fully understood the study’s purpose and procedures. All tissue samples obtained were confirmed as CRC and immediately frozen in liquid nitrogen.

### Cell culture

Human CRC cell line HCT116 was obtained from the Culture Collection of the Chinese Academy of Science (Shanghai, China) and cultured in RPMI-1640 medium supplemented with 10% fetal bovine serum (FBS) and 1% penicillin/streptomycin. HEK-293T cells were maintained in Dulbecco’s Modified Eagle Medium containing 10% FBS. All cell lines were grown in a 37°C incubator with 5% CO_2_. The cell lines were authenticated using short tandem repeat authentication by Genetic Testing Biotechnology Corporation and were also screened for mycoplasma contamination.

### Bioinformatics analysis

The database UALCAN was used to explore the expression levels of NOL6 in COAD and READ based on individual cancer stages. The survival analysis curve between the high and low expression of NOL6 groups were obtained from the Kaplan-Meier plotter.

### Lentiviral packaging and knockdown cell line construction

Lentiviral-mediated transduction was used to introduce synthetic short hairpin RNA (shRNA) constructs to specifically target NOL6, along with non-targeting (control) oligonucleotides. The RNA interference target sequences were carefully designed to selectively bind to the NOL6 gene. The selected interference targets, NOL6 shRNA1 (psc42498) and shRNA2 (psc42500), had the sequences 5′-GCTAGTAATGGTGCCCAAT-3′ and 5′-TCGGATTGATGCCTTCCTA-3′, respectively. These NOL6-targeting shRNA duplexes and the negative control shRNA were synthesized by Shanghai Genechem Co., Ltd., to create positive recombinant constructs, following the manufacturer’s protocol. The accuracy of the insert sequences was verified, and the plasmids were purified for subsequent use. Recombinant adenoviruses were produced using homologous recombination and cultured in 293T cells. The virions were harvested from the cell culture supernatant using density gradient centrifugation and stored at −80°C for future analysis.

To establish a stable NOL6 knockdown cell line, HCT116 cells were infected with the shRNA1 (psc42498), shRNA2 (psc42500), and shNC lentiviral particles. The cells were then selected using 10 μg/ml puromycin to ensure only cells with the integrated shRNA constructs survived.

### Quantitative real-time PCR (qRT-PCR)

Total RNA was extracted from the cells using the TRIzol reagent (SuperfecTRI). The quality and quantity of the extracted RNA were assessed using a NanoDrop spectrophotometer (Thermo Fisher Scientific). One micogram of total RNA was reverse transcribed into complementary DNA (cDNA) using the Reverse Transcription Kit (Promega M-MLV), following the manufacturer’s protocol. Subsequently, qRT-PCR was conducted using a SYBR Green PCR master mix on a LightCycler 480 instrument based on our previous research. The thermal cycling conditions included an initial denaturation step (at 95°C for 10 min), followed by 45 amplification cycles of denaturation (at 95°C for 10 s), annealing (at 60°C for 30 s), and extension (at 72°C for 30 s). Glyceraldehyde 3-phosphate dehydrogenase (GAPDH) was used as an endogenous control, and the relative expression levels of NOL6 were calculated using the 2^−ΔΔCT^ method. The specific primer pairs for NOL6 were 5’-TGAGGCACGGCTGTCTATGAT-3’ (forward) and 5’-GGAGATGCAGGACATGGTC-3’ (reverse).

### Immunohistochemistry (IHC) analysis

Protein expression levels in tissue sections obtained from the First People’s Hospital were analyzed using IHC from 2024.12.1–2025.5.30. The IHC analysis was conducted as previously described, with a slight modification [11,16]. Tissue sections were deparaffinized with xylene, rehydrated with a graded alcohol series, and washed with distilled water. The sections were then incubated overnight with rabbit-anti NOL6 primary antibody (NBP1–57231, Novus Bio) and treated with a secondary antibody conjugated with horseradish peroxidase (HRP) for 1 h. The sections were counterstained with hematoxylin, dehydrated, and mounted on slides. Protein localization and expression levels of NOL6 were examined and quantified under a microscope.

### Western blot

Fresh cells were washed with phosphate-buffered saline (PBS) solution and lysed with RIPA buffer supplemented with PMSF for 30 min on ice to obtain total protein. The protein concentration was determined using a BCA protein assay kit (Beyotime Biotechnology). A 10% SDS-PAGE gel was used to separate 30 μg of protein from the lysate, and then the proteins were transferred to a PVDF membrane (Millipore) using transfer electrophoresis at 4°C (300 mA, 150 min). The membranes were then incubated overnight with the specified primary antibodies at 4°C, followed by incubation with HRP-conjugated secondary antibodies for 1 h at room temperature. Target proteins were visualized on the membrane using a 20**×** LumiGLO^®^ Reagent and 20**×** Peroxide kit.

### Colony formation assay

Following lentiviral transfection, HCT116 cells in the logarithmic growth phase were resuspended to form a uniform cell suspension and counted to determine the total number of cells. The transfected cells were then seeded into a six-well plate at a density of 1,000 cells per well, with triplicate wells established for each experimental condition. The seeded cells were cultured in an incubator set to 37°C with 5% CO_2_ for 2 weeks. The culture medium was refreshed as required throughout the incubation period, and the cells were monitored every 3 days to assess their conditions. Following a 14-day incubation period, the cell colonies were visualized using a microscope (Olympus), and their images were documented for further analysis. Subsequently, the cells were rinsed once with PBS, fixed with a 4% paraformaldehyde solution, and stained with GIEMSA to visualize the colonies. After staining, the cells were thoroughly washed with double-distilled water (ddH_2_O) and air-dried. Colony formation was photographed, and the number of colonies in each group was quantified.

### Cell viability assay

Transfected HCT116 cells were seeded in 96-well plates at a density of 2,000 cells per well and cultured at 37°C in an atmosphere containing 5% CO_2_. Beginning from the day after inoculation, 20 μL of 5 mg/mL MTT (Genview) was added to each well for 4 h. The supernatant was completely aspirated and discarded, followed by the addition of 100 mL DMSO (Shanghai Shiyi Chemicals Reagent) to each well. The plates were gently oscillated for 2–5 min to ensure complete dissolution of the crystals. The absorbance of each well was measured, and the optical density (OD) values were obtained at 490 nm using an enzyme-linked microplate reader (Tecan Infinite). Each time point was assayed in triplicate, and the experiment was replicated three times.

### Celigo® cell count assay

HCT116 cells in logarithmic growth period were collected and digested with 0.05% pancreatic enzyme and suspended in RPMI 1640 medium, followed by cell counting. HCT116 cells (100 µl; 1 × 10^6^ cells/ml) were added to each well of a 6-well plate and cultured at 37°C with 5% CO_2_. Subsequently, the Celigo^®^ assay was performed every day for 5 consecutive days.

### Migration and invasion assays

During the logarithmic growth phase, shNOL6 and shNC cells were harvested using trypsin and resuspended in a complete growth medium to form a cell suspension. In the transwell migration assay, transwell chambers were placed in a 24-well culture plate. The lower compartment was prefilled with 750 μl of RPMI-1640 medium supplemented with 30% FBS. A suspension containing 1 × 10^5^ cells in 500 μl of serum-free medium was added to the upper chamber. After a 72-h incubation period, the cells that had migrated to the lower compartment were fixed, stained with crystal violet, and photographed for quantification.

For the invasion assay, the upper chamber was coated with precooled Matrigel (Corning, USA) before cell seeding, and the experimental protocol followed a similar process to that of the migration assay.

### Flow cytometry for cell cycle analysis

First, cells from six-well plates (>**1×**10^6^) were harvested and washed twice with cold 1 × Hanks’ balanced salt solution (HBSS). The harvested cells were fixed using 75% ethanol and stored for at least 1 h for cell cycle analysis. After rehydration in PBS, the cells were treated with 20 μl of RNase A (2 μg/ml) and incubated at 37°C for 30 min. Incubated cells were then stained with propidium iodide (PI, 50 μg/ml) for 1 h at 4°C. The stained cells were then resuspended in a staining buffer and incubated in the dark at room temperature. After incubation, the cells were analyzed using a Millipore Guava cytometer (Millipore), and the data were analyzed using Millipore’s InCyteTM Software (Millipore).

### Animal models

BALB/c nude mice were randomly assigned to two experimental groups, each comprising 10 animals. All BALB/c nude mice were housed in a temperature- and humidity-controlled vivarium on a 12-hour dark-light cycle with free access to food and water. HCT116 cells in the logarithmic phase of growth were harvested, resuspended to form a homogeneous cell suspension, and counted post-lentiviral transfection. These mice were then inoculated with 5 × 10^6^ stable transfected HCT116 cells suspended in 200 μl of PBS in the right armpit region. After one week of injections, tumor volume was measured every two days. The long (L) and short (W) diameters of the tumor were measured using a Vernier caliper. The tumor volume (V) was calculated using the formula: V = π/6 × L × W^2^. No mice died during the experiment before meeting criteria for euthanasia. Two weeks post-inoculation , the mice were anesthetized through intraperitoneal injection of a 3% pentobarbital solution at a dosage of 160 mg/kg and then euthanized. The tumors were carefully excised from the inoculation site, and the weights of the excised xenograft tumor tissues were statistically analyzed to assess differences between the experimental groups. All experimental procedures involving animals were performed in accordance with the Guide for the Care and Use of Laboratory Animals (NIH publications Nos. 80–23, revised 1996) and the Institutional Ethical Guidelines for Animal Experiments and were approved by the Ethical Committee of the First People’s Hospital of Chenzhou City, university of South China.

### TMT proteomic analysis

HCT116 cells infected with shNOL6 or shNC for 72 h were used for protein extraction. Proteins were extracted using SDT lysis buffer and quantified using the BCA protein assay kit. The extracted and quantified protein samples were labeled using the TMT Labeling Kit (Thermo Fisher Scientific, USA). The labeled samples were mixed in equal proportions and separated using a High pH Reversed-Phase Peptide Fractionation Kit. The peptide fractions were further separated using an Easy-nLC High-Performance Liquid Chromatography (HPLC) system (Thermo Fisher Scientific, USA) at a nanoliter flow rate. The separated samples were stored until further analysis.

### Protein data and bioinformatics analysis

Separated samples were analyzed using a Q Exactive plus mass spectrometer (Thermo Fisher Scientific, USA). The original mass spectrometry data were generated as RAW files. Proteins present in the samples were identified using Proteome Discoverer 2.1 (Thermo Fisher Scientific) and quantified using Mascot 2.6. A fold change > 1.2 or < 0.83 and a p-value < 0.05 (*t* test) were considered significan*t* between two comparable groups (between the NOL6-knockdown groups and control groups). The significantly differentially abundant proteins were subjected to various bioinformatics analyses including pathway enrichment analysis, subcellular localization analysis, Gene Ontology (GO) analysis, and Kyoto Encyclopedia of Genes and Genomes (KEGG) analysis.

### Statistical analysis

All statistical analyses were performed using the SPSS and GraphPad Prism software. Data are presented as the mean ± Standard Error of the Mean (SEM) or median (quartile), depending on the data distribution. Significant differences in paired and unpaired continuous variables were assessed using Student’s *t* test or the Wilcoxon signed-rank test, respectively. For unpaired data comparisons, a two-tailed Student’s *t* test or one-way ANOVA was employed. Differences in the expression or distribution of categorical variables were analyzed using Pearson’s chi-square (*χ*^2^) test or Fisher’s exact test, as appropriate. The Kaplan-Meier method, log-rank test, and univariate Cox proportional hazard regression analysis were performed to estimate potential prognostic indicators. Each experiment was performed in three replicates to ensure the reliability of the results. All p-values were two-tailed, and a threshold of p < 0.05 was set to determine statistical significance for all tests.

## Results

### Expression of NOL6 was upregulated in CRC tissues

**Fig 1:**
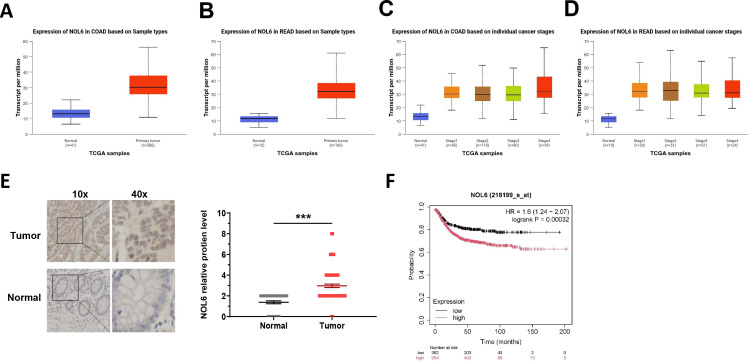
NOL6 was highly expressed in colorectal cancer cells. **(A)** The expression of NOL6 is increased in the colon adenocarcinoma (COAD) compared to the normal tissues. **(B)** The expression of NOL6 is increased in the colon adenocarcinoma rectal adenocarcinoma (READ) compared to the normal tissues. **(C)** Expression levels of COAD were explored based on individual cancer stages. **(D)** Expression levels of READ were explored based on individual cancer stages. **(E)** IHC score of NOL6 in cancerous tissue. Left, therepresentative image; right, the statistical result. Error bars represent SEM. **(F)** Kaplan–Meier curve of OS of COAD were explored based on the expression of NOL6 by KM-plot. *p < 0.05, **p < 0.01, ***p < 0.005, ****p < 0.001.

To determine NOL6 expression levels in CRC tissues, we analyzed data from the CRC cohort in The Cancer Genome Atlas (TCGA) database. The result showed a significant upregulation of NOL6 in CRC tissues compared with normal controls by UALCAN (Fig 1A-B). Further analysis found the different expression levels of NOL6 in different colorectal cancer stages (Fig 1C-1D). Additionally, IHC was used to assess NOL6 expression in 97 CRC samples and paired adjacent non-tumor tissues. The results revealed a significant increase in NOL6-positive staining in cancer tissues compared with normal colorectal tissues (Fig 1E). Moreover, we found the patients that have higher NOL6 expression levels have a poorer prognosis than the lower by the KM-plot ((Fig 1F). These findings collectively demonstrate that NOL6 is upregulated in CRC tissues.

### NOL6 knockdown suppressed cell proliferation and arrested the cell-cycle progression in CRC cells

**Fig 2:**
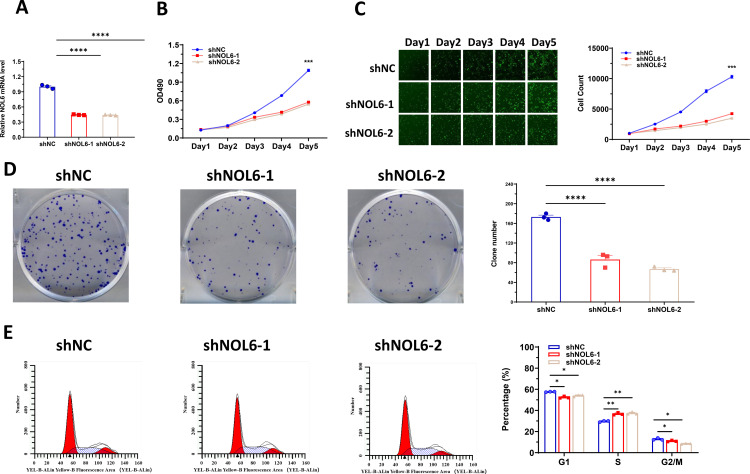
NOL6 knockdown suppressed cell proliferation and arrested cell-cycle progression in CRC cells. **(A)** RNA expression of HCT 116 cells infected with shNOL6 and shNC was assessed using qRT-PCR. **(B)** NOL6 knockdown suppressed the cell proliferation ability of HCT116 cells, as determined using MTT assay. **(C)** NOL6 knockdown inhibited the growth of HCT116 cells as demonstrated by the fluorescence imaging system Celigo. Left, the fluorescence image; right, the statistical result. **(D)** NOL6 knockdown suppressed colony formation in HCT116 cells. Left, the representative image of cell colonies; right, the statistical result. **(E)** NOL6 knockdown arrested cell-cycle progression in CRC cells. Left, the representative image; right, the statistical result. Error bars represent SEM. *p < 0.05, **p < 0.01, ***p < 0.005, ****p < 0.001.

To evaluate the effect of NOL6 on the proliferative capacity of CRC cell lines, HCT116 cells were transduced with lentivirus expressing shNOL6 and shNC. qRT-PCR analysis indicated a significant decrease in NOL6 mRNA expression levels in the shNOL6 group compared with the control group (Fig 2A) following shRNA treatment. MTT assay revealed that the cell proliferation rates of the NOL6-shRNA group were significantly lower than those of the control group (Fig 2B). Furthermore, cell proliferation was assessed using the Celigo imaging cytometer (Nexcelom). The results revealed that NOL6 knockdown inhibited the proliferation of HCT116 cells (Fig 2C). Additionally, colony formation assay indicated that the number of colonies formed by HCT116 in the NOL6-shRNA group was significantly lower than that of the control group (Fig 2D). These results suggest that silencing NOL6 suppresses the proliferation and tumorigenesis of CRC cells.

To determine the relationship between the inhibitory effect of NOL6 on CRC cell proliferation and cell cycle arrest, a flow cytometry analysis was performed. The apoptotic rates of the shNOL6 and shNC groups were also determined using flow cytometry. As illustrated in Fig 2E, the proportion of cells in G1 phase (shNOL6−1: 52.3% ± 0.96% vs. 57.6% ± 0.30%, p < 0.05; shNOL6−2: 54% ± 0.86% vs. 57.6% ± 0.30%, p < 0.05), S phase (shNOL6−1: 36.7% ± 1.11% vs. 29.67% ± 0.62%, p < 0.05; shNOL6−2: 37.4% ± 1.21% vs. 29.7% ± 0.62%, p < 0.05) and G2/M (shNOL6−1: 11.0% ± 0.70% vs. 12.8% ± 0.92%, p ＝ 0.05; shNOL6−2: 8.57% ± 0.35% vs. 12.8% ± 0.92%, p < 0.05), compared to control group. These results revealed that the shNOL6 group cells increased significantly in S phase, and decreased in G1 phase.

### NOL6 knockdown suppressed cell migration and invasion in CRC cells

**Fig 3:**
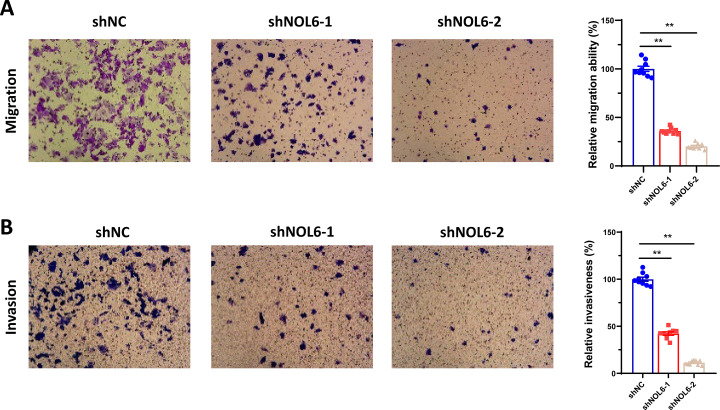
NOL6 knockdown inhibited the migration and invasion of HCT116. **(A)** Transwell migration assays demonstrated that NOL6 knockdown inhibited the migration of HCT116 cells (magnification ×200). **(B)** Transwell migration assays indicated that NOL6 knockdown inhibited cell migration (magnification ×200). Left, the representative image; right, the statistical result. Error bars represent SEM. *p < 0.05, **p < 0.01, ***p < 0.005, ****p < 0.001.

To determine the role of NOL6 in the migratory and invasive behavior of CRC cell lines, we examined the invasive potential of HCT116 cells after NOL6 knockdown using the transwell assay, following the manufacturer’s protocol. The results indicated that the number of migrating and invading HCT116 cells was significantly lower in the shNOL6 group than in the shNC group (Fig 3), suggesting that NOL6 knockdown inhibited cell migration and invasion in CRC cells.**NOL6 knockdown inhibited tumor growth in vivo**

**Fig 4:**
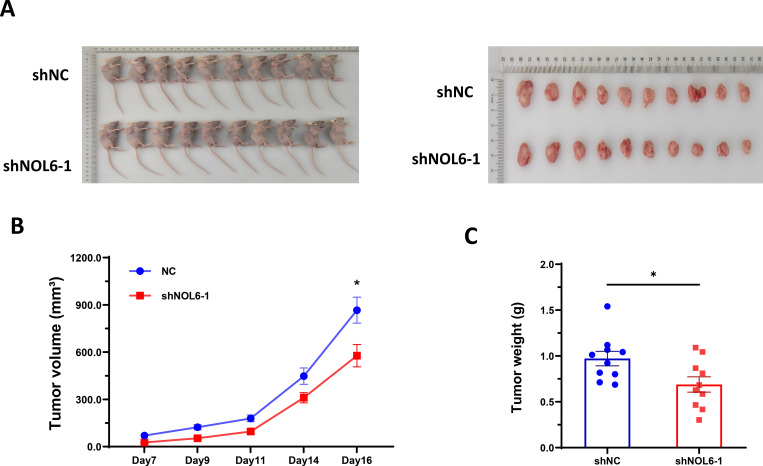
NOL6 knockdown inhibited the growth of CRC cells *in vivo.* **(A)** NOL6 knockdown inhibited subcutaneous tumor formation in a nude mouse model. **(B)** NOL6 knockdown decreased tumor weight. **(C)** NOL6 knockdown decreased tumor growth *in vivo*. Error bars represent SEM. *p < 0.05, **p < 0.01, ***p < 0.005, ****p < 0.001.

To investigate the inhibitory potential of NOL6 knockdown on CRC tumor growth, we conducted *in vivo* animal experiments. The results demonstrated that NOL6 inhibition significantly decreased the volume and weight of tumors compared with the shNC group (Fig 4).

### NOL6 regulated distinct gene expression in CRC

**Fig 5:**
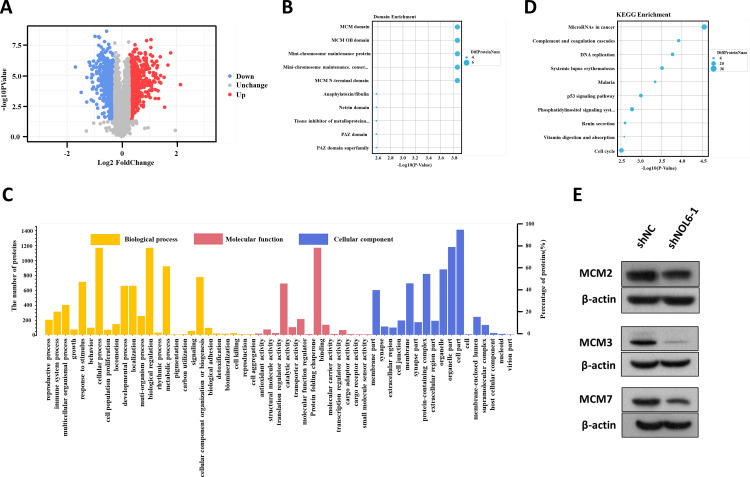
NOL6 regulated distinct gene expression in CRC. **(A)** Volcano plot illustrating protein quantification and differential analysis. **(B)** Annotation and enrichment of functional domains of differentially expressed proteins. **(C)** Enrichment analysis of differentially expressed proteins according to GO terms, including biological process, cellular component, and molecular function; **(D)** pathway classification using KEGG enrichment. The x-axis represents protein number, and the y-axis represents KEGG pathway enrichment; **(E)** expression levels of MCM2, MCM3, and MCNA across different groups.

To determine the underlying mechanisms by which NOL6 influences CRC, we employed TMT-labeled quantitative proteomic technology and bioinformatic analysis. A total number of 1,501 differentially expressed genes, which comprised 828 upregulated and 673 downregulated genes, were identified in the NOL6 knockdown group compared with the shNC group, as shown in the volcano plot (Fig 5A). Domain enrichment analysis showed that the differentially expressed genes were significantly enriched at MCM domain, MCM OB domain, and Mini-chromosome maintenance protein (Fig 5B). KEGG pathway analysis revealed that these proteins were significantly enriched in several pathways, including the microRNA pathway in cancer, complement and coagulation cascades, and DNA replication pathway in eukaryotes (Fig 5C). GO analysis further revealed that the differentially expressed proteins were primarily associated with the extracellular space, MCM complex, and platelet alpha granule lumen (Fig 5D). These findings reveal the potential role of NOL6 in regulating the expression of MCM family genes.

Following the NOL6 knockdown, the genes MCM2, MCM3, and MCM7 were analyzed and identified as downstream elements in the gene interaction network and were selected for further investigation. The protein expression levels of MCM2, MCM3, and MCM7 were assessed using Western blotting assays. The results showed no significant change in MCM2 protein expression levels; however, MCM3 and MCM7 protein levels were significantly downregulated following NOL6 knockdown (Fig. 5E). These findings suggest that NOL6 knockdown suppressed MCM3 and MCM7 expression.

## Discussion

NOL6 has been identified as a potential oncogene in various cancers, including prostate, gastric, and hepatocellular carcinomas [17–20]. However, its specific role in CRC is not fully understood. Given its potential regulatory functions in other cancer types, further investigation into the role of NOL6 in CRC is required. This study found that NOL6 was upregulated in CRC tissues, and its knockdown inhibited cell growth both *in vitro* and *in vivo*. Mechanistically, NOL6 knockdown may suppress MCM3 and MCM7 expression.

Although several methodologies have been developed for diagnosing CRC, the identification of classical oncogenes, such as APC, TP53, KRAS, and BRAF, has been instrumental in providing diagnostic biomarkers [21–25]. Notably, certain genes, such as KRAS and BRAF, have emerged as potential indicators of the efficiency of novel targeted therapies [26,27]. Despite significant advancements in surgical interventions and multimodal treatment strategies, the prognosis for CRC patients remains poor, primarily due to late diagnosis [23,28]. Therefore, further investigation into the genetic mechanisms underlying CRC is essential, as it could reveal more prognostic markers, molecular predictive factors, and determinants for targeted therapy efficacy.

Studies have associated NOL6 nucleolar localization with ribosomal biogenesis. This gene encodes proteins associated with nucleolar RNA and is highly conserved across various species [17,18]. A study by Lin et al. demonstrated that the suppression of NOL6 expression in cells resulted in G1 phase arrest and facilitated cell death [29]. These findings highlight the role of NOL6 in rRNA processing and cell-cycle progression regulation.

This study is the first to demonstrate the regulatory role of NOL6 in the development of CRC. Sample data analyzed from TCGA database revealed elevated NOL6 expression levels in CRC tissues compared with adjacent tissues. Additionally, a significant correlation was observed between NOL6 expression and the pathological TNM stage, suggesting its role in CRC progression. Furthermore, NOL6 knockdown resulted in suppressed cell proliferation, decreased colony-forming ability, and arrested cell-cycle. Notably, the weight and volume of tumors in shNOL6 mice were significantly lower, which was consistent with our *in vitro* study. Therefore, we hypothesized that NOL6 plays a crucial role in the initiation and progression of CRC. Advancements in sequencing technology now allow a more comprehensive analysis of the interplay between genes and diseases. In this study, we identified potential downstream targets following NOL6 knockdown. The results revealed MCM2, MCM3, MCM7, and PCNA as integral components of the NOL6 network. Western blot analysis further confirmed that NOL6 inhibition downregulated MCM3 and MCM7 expression, validating the findings and the reliability of the results. Elevated expression levels of the MCM family genes, specifically MCM2, MCM3, and MCM7, were significantly correlated with advanced clinical cancer stages. MCM3 and MCM7 were associated with adverse clinical outcomes and played a crucial role in facilitating G1/S cell cycle transition, proliferation, migration, and invasion in CRC. Notably, increased mRNA expression of MCM3 and MCM7 was associated with prolonged overall survival. Furthermore, higher mRNA expression levels of MCM2, MCM3, and MCM7 were linked to a more favorable overall survival rate [30,31].

The tumor microenvironment and immune status are critical determinants of tumor prognosis. Recent studies have highlighted the role of circNOL6A3 in regulating immune escape in colorectal cancer, where its knockout was shown to reduce myeloid-derived suppressor cells (MDSCs) and increase CD8 ⁺ T cell infiltration [32]. Additionally, the gut microbiota can modulate tumor treatment responses by influencing both the tumor microenvironment and drug efficacy [33,34]. Certain natural products have also demonstrated the ability to reverse decitabine resistance and remodel the tumor microenvironment, thereby enhancing therapeutic outcomes [35]. Although the functions of NOL6 in the tumor microenvironment, drug resistance, and gut microbiota remain unclear, further investigation into its role may reveal novel candidate targets for cancer therapy.

In summary, our findings suggest that NOL6 functions as a potential oncogenic factor in CRC by inducing the expression of MCM2 and MCM7. Therefore, NOL6 may serve as a biomarker and therapeutic target for CRC.

## Supporting information

S1 File Raw data. (XLS)
